# Rapid crowdsourced innovation for COVID-19 response and economic growth

**DOI:** 10.1038/s41746-021-00397-5

**Published:** 2021-02-09

**Authors:** Khalil B. Ramadi, Freddy T. Nguyen

**Affiliations:** 1 Hacking Medicine, Massachusetts Institute of Technology, Cambridge, MA USA; 2 School of Engineering, Massachusetts Institute of Technology, Cambridge, MA USA; 3 Harvard TH Chan School of Public Health, Harvard University, Boston, MA USA; 4 Division of Engineering, New York University Abu Dhabi, Abu Dhabi, UAE; 5 Tandon School of Engineering, New York University, New York, NY USA; 6 Innovation Initiative, Massachusetts Institute of Technology, Cambridge, MA USA; 7 Institute for Medical Engineering and Sciences, Massachusetts Institute of Technology, Cambridge, MA USA; 8 Icahn School of Medicine, Mt Sinai Hospital, New York, NY USA

**Keywords:** Health care, Biomedical engineering

## Abstract

The COVID-19 pandemic has profoundly affected life worldwide. Governments have been faced with the formidable task of implementing public health measures, such as social distancing, quarantines, and lockdowns, while simultaneously supporting a sluggish economy and stimulating research and development (R&D) for the pandemic. Catalyzing bottom-up entrepreneurship is one method to achieve this. Home-grown efforts by citizens wishing to contribute their time and resources to help have sprouted organically, with ideas shared widely on the internet. We outline a framework for structured, crowdsourced innovation that facilitates collaboration to tackle real, contextualized problems. This is exemplified by a series of virtual hackathon events attracting over 9000 applicants from 142 countries and 49 states. A hackathon is an event that convenes diverse individuals to crowdsource solutions around a core set of predetermined challenges in a limited amount of time. A consortium of over 100 partners from across the healthcare spectrum and beyond defined challenges and supported teams after the event, resulting in the continuation of at least 25% of all teams post-event. Grassroots entrepreneurship can stimulate economic growth while contributing to broader R&D efforts to confront public health emergencies.

## Introduction

The COVID-19 pandemic has drastically disrupted daily life. Over the course of a few months, people’s movements have been restricted, businesses forced to close and health systems pushed to new limits. The pandemic, however, has on an unprecedented level focused the world’s attention on a single common problem. The explosion of home-grown efforts to address the crisis, ranging from designs of personal protective equipment (PPE) and home-made ventilators, to sourcing of materials and supplies from non-medical industries^1,2^ has turned individuals into problem solvers, innovators, and entrepreneurs. Top-down government initiatives to spur innovations have centered on financial incentives for existing entities and businesses to provide critical equipment and supplies, or to repurpose existing infrastructures towards new use cases. However, how do we foster an ecosystem that combines top-down with organic grassroots innovation? Early-stage entrepreneurship crowdsources solutions to multi-faceted problems presented by the pandemic and fosters economic growth in times of recession.

Implementation of bottom-up innovations for COVID-19 requires coordinated effort and early involvement of key stakeholders in the healthcare ecosystem, including patients, providers, payers, administrators, and public health experts. We describe a targeted multi-stakeholder effort to develop solutions for problems caused or exacerbated by the COVID-19 pandemic. This effort has brought together partners from across the healthcare spectrum and beyond, enabling rapid, iterative design to identify optimal approaches for a given problem and context^3^. Crowdsourcing is an emerging concept where solutions to problems are generated by a broad group of people^4^, and has been implemented for specific objectives related to COVID-19^5,6^. More specifically in healthcare, the World Health Organization (WHO) defines crowdsourcing as “the process of having a large group solve a problem and then share solutions with the public”^7^. Healthcare hackathons (Box 1) utilize this to facilitate the development of new health innovations^8,9^.

The Massachusetts Institute of Technology (MIT) COVID-19 Challenge was launched in mid-March 2020 as a series of virtual hackathon events focused on addressing immediate needs with an eye towards rapid innovation from ideation to implementation and impact. These events built on an established methodology to achieve a number of goals: (1) contextualize an impactful problem to a specific real-life scenario, (2) convene cognitively, geographically, and socially diverse teams to tackle the problem, and (3) assemble an equally diverse set of domain and process expert mentors to provide accelerated customer discovery and subject matter expertize, and (4) optimize the design for accelerated development and implementation.

**Fig. 1:**
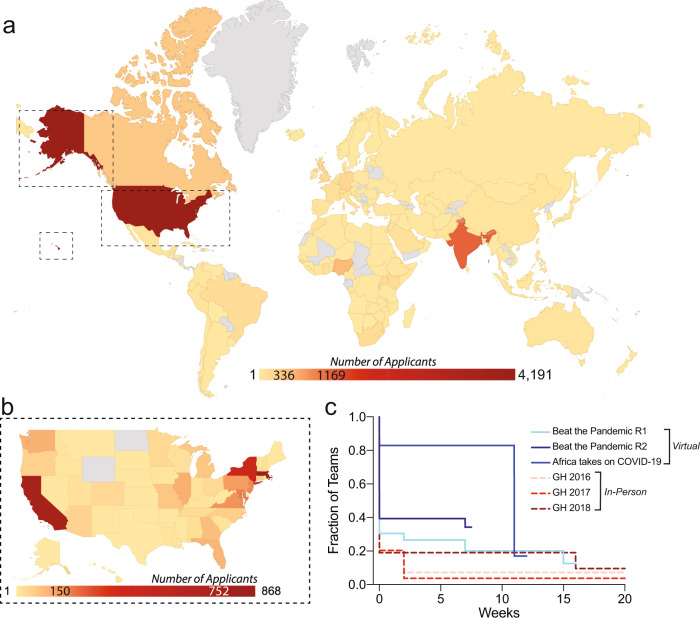
Worldwide crowdsourced innovation. **a**, **b** Number of applicants per (**a**) country around the world and (**b**) state in the US for MIT COVID-19 Challenges. Gray color shading corresponds to no applicants from that country. **c** Fraction of teams continuing to work on their ideas post-event. Plots show team continuation for teams from recent virtual events and in-person events in 2016, 2017, and 2018 (GH, Grand Hack). Maps generated using Google GeoChart API under Creative Commons Attribution 4.0 License.

The MIT COVID-19 Challenge utilized the healthcare hackathon in a virtual, rather than in-person, event. The virtual platform multiplied the scale and reach beyond what would have been possible with traditional in-person events, by removing financial, time, and travel barriers. The largest in-person healthcare hackathons (such as MIT Hacking Medicine’s yearly Grand Hack) typically include approximately 400 participants and 100 mentors. By comparison, the “Beat the Pandemic” (BTP) hackathons (Round 1, April 3–5, 2020; Round 2, May 29–31 2020) and “Africa Takes on COVID-19” challenge (May 1–3, 2020) combined over 9000 applicants from 142 countries and 49 states (Fig. 1a, b, Table 1) for <1500 participant slots per event, and 1000+ mentor applications for ~250 mentor slots per event even in recruitment periods of less than 2 weeks. Applicants represented health, science, engineering, policy, design, business, finance, and regulatory disciplines (Table 2). Approximately 35.9% of applicants were from low and middle-income countries (LMICs). Each event facilitated the organic formation of approximately 200 teams spread across 10 tracks, each with challenges identified in conjunction with partners and stakeholders. An average of 70 partners were recruited for each event ranging from universities, hospitals, and health systems to incubators, accelerators, venture capital, technology companies, biotech, and pharmaceutical companies. Additional relevant stakeholders to the African continent included NGOs (e.g., Clinton Foundation, ONE Campaign, United National Development Program) and public health agencies (e.g., Nigerian Centre for Disease Control (CDC)).

**Table 1:** Total number of applicants by country across three MIT COVID-19 Challenge events.

Country	No.	Country	No.	Country	No.	Country	No.
Afghanistan	1	Djibouti	1	Libya	8	Slovenia	7
Albania	5	Dominican Republic	3	Macedonia	1	Somalia	8
Algeria	18	Ecuador	27	Madagascar	3	South Sudan	1
Angola	1	Egypt	21	Malawi	17	Spain	49
Argentina	12	Eritrea	1	Malaysia	13	Sri Lanka	12
Armenia	1	Estonia	2	Mauritania	1	Sudan	6
Australia	49	Eswatini (fmr. “Swaziland”)	2	Mauritius	5	Sweden	4
Austria	12	Ethiopia	35	Mexico	45	Switzerland	18
Bahamas	4	Finland	4	Mongolia	1	Syria	1
Bangladesh	15	France	102	Morocco	75	Taiwan	40
Belgium	9	Germany	200	Mozambique	13	Tanzania	18
Benin	2	Ghana	85	Myanmar (formerly Burma)	2	Thailand	5
Bhutan	1	Greece	8	Namibia	3	The Netherlands	1
Bolivia	5	Guatemala	2	Nepal	9	Togo	2
Bosnia and Herzegovina	1	Guinea	1	Netherlands	45	Trinidad and Tobago	3
Botswana	5	Guinea-Bissau	1	New Zealand	6	Tunisia	22
Brazil	125	Honduras	2	Niger	2	Turkey	24
Brunei	1	Hungary	6	Nigeria	482	Turkmenistan	1
Bulgaria	1	Iceland	1	Norway	3	Uganda	161
Burkina Faso	2	India	1169	Oman	1	Ukraine	6
Cabo Verde	1	Indonesia	38	Pakistan	50	United Arab Emirates	34
Cameroon	40	Iran	11	Palestine State	28	United Kingdom	190
Canada	336	Iraq	1	Peru	58	United States of America	4191
Chile	15	Ireland	13	Philippines	21	Uruguay	1
China	60	Israel	16	Poland	16	Uzbekistan	1
Colombia	26	Italy	84	Portugal	18	Vanuatu	1
Congo (Congo-Brazzaville)	2	Jamaica	2	Qatar	1	Venezuela	2
Costa Rica	3	Japan	22	Romania	11	Vietnam	8
Côte D’Ivoire	6	Jordan	1	Russia	9	Zambia	25
Croatia	1	Kazakhstan	14	Rwanda	26	Zimbabwe	30
Cuba	1	Kenya	157	Saudi Arabia	79		
Cyprus	1	Kuwait	1	Senegal	9		
Czech Republic	2	Latvia	2	Sierra Leone	16		
Czechia (Czech Republic)	3	Lebanon	14	Singapore	80		
Democratic Republic of the Congo	14	Lesotho	1	South Africa	141		
Denmark	11	Liberia	5	South Korea	13

**Table 2:** Number of applicants per discipline per event.

Discipline	BTP1	Africa	BTP2
Business/finance	589 (12.4%)	361 (15.7%)	271 (13.4%)
Data engineer/data scientist	456 (9.6%)	118 (5.1%)	219 (10.8%)
Designer	367 (7.7%)	103 (4.5%)	128 (6.3%)
Engineer	987 (20.7%)	382 (16.6%)	376 (18.6%)
Patient/patient’s family	10 (0.2%)	9 (0.4%)	13 (0.6%)
Clinician/provider	464 (9.7%)	316 (13.7%)	243 (12.0%)
Scientist	661 (13.9%)	283 (12.3%)	310 (15.3%)
Software	741 (15.5%)	334 (14.5%)	357 (17.6%)
Media	64 (1.3%)	61 (2.6%)	46 (2.3%)
Other (Health policy/Law/regulatory)	428 (9.0%)	338 (14.7%)	62 (3.1%)
Total	4767	2305	2025

Track themes were sourced from both event partners and participant applications, amplifying the most pressing needs based on the timing of each event using a hybrid top-down and bottoms-up approach. Some track themes tackled deployment and coordination of COVID-19 testing, triaging, education and resources. Other focused on novel care delivery paradigms, health supply allocation, and healthcare workforce well-being and training. Some themes such as COVID-19 testing and misinformation crossed multiple events and necessitated different solutions depending on the local ecosystem they would be implemented in. BTP events centered on COVID-19 issues in the developed world such as the United States, while “Africa takes on COVID-19” focused exclusively on the emerging needs associated with COVID-19 in Africa. Although events were not directly linked, some participants, mentors, and partners overlapped across the events. Each event had 10 tracks that participants could select to join, with each track focused on a distinct topic area^10^. Some continuing teams include WePool^11^ which focused on smart pooling of COVID-19 testing; SANIPACK which focused on the development of personal N-95 UV-C sterilizers^12^; and Birthing Bridge^12,13^ which focused on coordination of pre-natal care to pre-eclampsia patients in South African townships.

These programs achieve multiple objectives. Coordination of innovation efforts across individuals and entities from around the world break down social, cultural, and institutional barriers to multi-disciplinary collaborations^14^. Organized crowdsourcing leverages diverse skillsets to produce highly viable concepts and prototypes to quickly tackle problems^15^. The flat structure across participants, mentors, and partners, whereby intellectual property is not compromised, facilitates rapid development, iteration, and implementation.

Translation of these ideas requires an equal or greater emphasis on post-hackathon activities. Follow-on engagement opportunities were developed with partners to support teams to further advance and implement their ideas. These included, but were not limited to, a build-a-thon (a hackathon focused on building software or hardware) in partnership with Amazon Web Services (AWS), pitch sessions, mentorship, 1:1 dedicated check-ins, and funding. Teams and partners were paired in a semi-organic fashion to prioritize fit between team needs and available partner resources. This approach balances the need to identify and cultivate the most viable solutions with identifying the best teams suited to execute.

The primary limitation to implementing ideas developed in hackathons is team perseverance in continuing development of their projects. This virtual platform enabled collaborative innovation despite quarantine and social distancing constraints. Regular follow-up surveys were sent to all participants prompting them to report any updates on their projects. No incentive was given to respond, and non-responders were assumed to have abandoned their projects. Overall, more than 25% of all teams continue working five weeks post-event. These results are only a few weeks after the event, and it is likely that more teams will stop working on their projects as time progresses. However, this rate of continuing teams is demonstratively higher than that of similar in-person events (average of 19% two weeks post-event) (Fig. 1c). One possible rationale for higher continuation rate following virtual, rather than in-person, events could be that teams formed on a virtual basis start accustomed to working in a decentralized and asynchronous manner compared to in-person hackathons where live co-working is a prerequisite. A second contributing factor is the greater proportion of time participants had during the time of quarantine and lockdowns, with few competing demands such as work, school, or social activities. Team continuation is likely also enhanced due to ubiquitous impending urgency to tackle the ongoing pandemic. Engaging teams quickly post-event with sustained support from strategic partners likely also decreased the attrition rate of teams compared to prior in-person events.

This approach interweaves bottom-up innovations along the translation pipeline through key strategic partnerships. Critiques of hackathons argue they have limited economic impact, as a result of their short duration^16^. However, they do allow for a rapid burst of context-specific solutions to be created and implemented for and by individual communities, addressing health inequity^17^. By activating untapped potential, bottom-up or grassroots innovation is a vital complement to traditional top-down institutional and governmental initiatives^18,19^. This crowdsourced approach to solutions has been shown to be effective across domains in new idea generation^20,21^. Catalyzing entrepreneurship and lowering the barrier to entry from such initiatives can serve as an engine for economic growth^22^, an issue of special importance given the economic downturn the pandemic has caused^23^.

These programs will not necessarily yield a new vaccine or drug therapy and are not meant to replace traditional R&D undertaken by academia and industry. They do, however, enable interdisciplinary collaborations and empower a new population to tackle problems that the ever-changing world brings. These events teach individuals problem solving skills, entrepreneurship, collaborative teamwork, and communication skills while creating new communities of entrepreneurs with novel solutions and potential ventures. There is great need to not only continue to implement these types of programs but to develop more robust research methodologies to assess and optimize the role and effectiveness of grassroots innovation within the greater context of healthcare innovation. The world has a shared interest in overcoming this pandemic and its downstream effects, and everyone should be given the opportunity to play an active role.

Box 1. Hackathons and virtual event format Hackathons are events that assemble participants to tackle problems within specific topic areas. Hackathons typically occur over multiple days, and allow for dedicated, focused time when teams brainstorm and develop solutions. The MIT Hacking Medicine health hackathon model combines participants from diverse backgrounds including science, engineering, medicine, design, payers, pharma, and policy^3^. Participants form teams around specific problem statements, presented by individual participants or pre-defined by event organizers. Teams then characterize the problem, distilling it into distinct sub-problems. The team selects a single addressable problem and brainstorms potential solutions including hardware, software, or policy proposals. Solution development is guided by continuous feedback from mentors and organizers. Mentors have broad experience across academia, industry, government, global health, and entrepreneurship sectors. Finally, teams present their ideas to a judging panel. Team ideas are evaluated based on impact, innovation, implementation, feasibility, viability, and presentation.

The COVID-19 hackathons replicated this in a virtual setting. Partnerships were established across academic, public, and private sectors. Participants applied via an online application form. Participants were selected based on application caliber, individuals’ alignment with event goals, and diversity of the participant pool. The entire event was run over Zoom and Slack, with opening and closing remarks delivered via webinars and individual team Zoom meeting rooms. Mentors joined team rooms as needed to provide guidance. Slack enabled asynchronous and synchronous communications where each team had a dedicated channel to interact with each other, mentors, and organizers. Participants formed teams through a problem pitching session. After 48 h of hacking, final pitch presentations were run in ten parallel tracks. Winning teams were announced in a closing webinar. Cash prizes ($500 USD) were awarded to four teams in each track. All teams were eligible to receive ongoing post-event support and engagement with organizers and partners of the MIT COVID-19 Challenge.

## Data Availability

All data is available from the authors upon reasonable request.
